# Subjective dry eye symptoms and associated factors among the national general population in China during the COVID-19 pandemic: A network analysis

**DOI:** 10.7189/jogh.13.06052

**Published:** 2023-12-01

**Authors:** Xue Wang, Yibo Wu, Fangkun Zhao, Wei Sun, Chang Pang, Xuange Sun, Shuang Zang

**Affiliations:** 1 Department of Community Nursing, School of Nursing, China Medical University, Shenyang, China; 2 School of Public Health, Peking University, Beijing, China; 3 Department of Ophthalmology, The Fourth Affiliated Hospital of China Medical University, Shenyang, China; 4 Department of Gerontology and Geriatrics, The Second Affiliated Hospital of Shenyang Medical College, Shenyang, China

## Abstract

**Background:**

The coronavirus disease 2019 (COVID-19) pandemic has presented numerous challenges to public health, including the emergence of subjective dry eye symptoms among individuals. However, there is currently a dearth of comprehensive knowledge regarding the associated factors with subjective dry eye symptoms in the general population.

**Methods:**

A nationwide survey in China was carried out from 20 June to 31 August 2022, involving 21 916 participants. Subjective dry eye symptoms were assessed using the Ocular Surface Disease Index-6. We employed random forest classification to select potential associated factors based on the socioecological model. We also conducted univariate and multivariable logistic regression analyses to explore the factors associated with subjective dry eye symptoms. Network analyses were employed to explore the network structure of subjective dry eye symptoms and associated factors.

**Results:**

The item “watching TV (or similar task)” displayed the highest node strength and exhibited the highest predictability within the network. The results of the multivariate logistic regression analysis demonstrated significant associations between subjective dry eye symptoms and several factors, including loneliness (odds ratio (OR) = 1.29; 95% confidence interval (CI) = 1.26 to 1.32), problematic Internet use (OR = 1.08; 95% CI = 1.08 to 1.09), family communication (OR = 1.01; 95% CI = 1.00 to 1.01), the presence of depression (OR = 1.53; 95% CI = 1.39 to 1.68) and anxiety (OR = 1.31; 95% CI = 1.17 to 1.47) symptoms, older age (OR = 1.01; 95% CI = 1.01 to 1.01), self-rated health status (OR = 0.99; 95% CI = 0.98 to 0.99), family health (OR = 0.97; 95% CI = 0.96 to 0.97), health literacy (OR = 0.98; 95% CI = 0.97 to 0.99) and the agreeableness personality trait (OR = 0.97; 95% CI = 0.95 to 0.99).

**Conclusions:**

These findings have important implications for public health interventions targeting the prevention and management of subjective dry eye symptoms in the general population. Strategies addressing individual risk factors and supporting psychological well-being may prove beneficial in mitigating the burden associated with subjective dry eye symptoms.

Dry eye symptoms are multifactorial ocular surface diseases characterised by an imbalance in tear film homeostasis [1]. These symptoms are accompanied by various ocular symptoms and involve factors such as tear film instability, hyperosmolarity, ocular surface inflammation and damage, and neurosensory abnormalities, all of which contribute to their etiology [2]. Dry eye symptoms are prevalent among individuals of diverse age groups and can significantly affect their quality of life and visual performance [3]. Gaining a comprehensive understanding of the underlying factors associated with dry eye symptoms is essential for the development of effective strategies for management and prevention.

The coronavirus disease 2019 (COVID-19) pandemic has brought about substantial changes in individuals’ daily routines and behaviours, with implications for ocular health [4,5]. The widespread implementation of online offices, online shopping, online education, increased reliance on digital devices, and social distancing measures have resulted in prolonged screen time and altered visual habits [6]. These changes have raised concerns regarding the potential exacerbation of dry eye symptoms during the pandemic [7]. Previous studies have identified some factors associated with the development and severity of dry eye symptoms, including demographic, psychological, and lifestyle factors [8,9]. For example, existing research has indicated that a patient’s personality traits can have some influence on subjective symptoms to a certain extent [10]. Personality traits may serve as the foundation for various psychological factors, which may play a role in the expression and perception of dry eye symptoms. In particular, previous study has shown a significant positive correlation between an individual’s neuroticism personality trait and dry eye disease [11]. In addition, the health levels of one’s family may be associated with an individual’s lifestyle and health habits, which can, to a certain extent, affect the occurrence of dry eye symptoms [12,13]. However, comprehensive investigations on the associations between subjective dry eye symptoms and the multitude of factors involved are lacking, particularly within the context of a holistic framework, such as the socioecological model.

The socioecological model encompasses five levels, namely individual characteristics, individual behaviours, interpersonal networks, community, and policy [14]. These levels collectively contribute to providing a holistic framework for comprehending health-related phenomena and evaluating factors associated with events from multiple perspectives [15,16]. This model recognises the reciprocal interactions between these factors and highlights the importance of considering the broader context in which dry eye symptoms occur. Applying the socioecological model to the study of subjective dry eye symptoms during the COVID-19 pandemic can offer a comprehensive understanding of the multifaceted factors associated with subjective dry eye symptoms. Building on previous studies [15,16], our research includes variables corresponding to each of these five levels: individual characteristics (i.e. age, gender, education level, personality traits, and health literacy), individual behaviours (i.e. smoking status, drinking alcohol, depression symptoms, anxiety symptoms, loneliness, sleep time, sleep quality, problematic Internet use, and self-rated health status), interpersonal networks (i.e. have a spouse, family health, and family communication), community (i.e. career status, urban-rural distribution, family per capita monthly income, and family social status), and policy (i.e. medical insurance type).

In addition, network analysis is a powerful methodology in complex systems research that enables the examination of interconnected associations among variables within a network framework [17]. By utilising network analysis, we can identify key factors and their strengths of association in the network of subjective dry eye symptoms. This approach allows for a comprehensive exploration of the intricate associations between factors within the socioecological model and their relative importance of subjective dry eye symptoms.

Therefore, this study aims to investigate the associations between subjective dry eye symptoms and factors within the framework of the multilevel socioecological model during the COVID-19 pandemic. By employing network analysis, we aim to identify the key factors and their interconnections, providing an understanding of the potential associations for subjective dry eye symptoms in the national general population.

## METHODS

### Survey design and participants

Between 20 June and 31 August 2022, we executed a survey in China, employing a multistage sampling design to guarantee the representativeness and generalisability of the sample. The survey spanned 23 provinces, five autonomous regions and four municipalities directly under the central government in China, representing 94.12% of the national population. It covered 148 cities, 202 districts and counties, 390 townships/towns/streets, and 780 communities/villages. The selection of respondents from the sampled cities employed quota sampling, with gender, age and urban-rural distribution as the quota attributes, designed based on demographic data proportions obtained from the “Seventh National Population Census in 2021” [18]. Data for this survey were obtained through one-on-one interviews with participants utilising an electronic questionnaire administered via the Wenjuanxing platform. Inclusion criteria for study participants were as follows: participants were required to be Chinese nationals aged 12 years or older, who willingly participated in the study, possessed the cognitive capacity to comprehend each questionnaire’s item, and were capable of independently completing the questionnaires. For individuals who could think but were unable to independently complete the questionnaire, the investigators conducted interviews by offering assistance while ensuring non-interference with the responses. Exclusion criteria for study participants were as follows: individuals diagnosed with cognitive impairments or mental disorders, those involved in concurrent similar studies, and those who dropped out of the study midway. Individuals diagnosed with mental health conditions by a qualified medical practitioner underwent comprehensive assessments that incorporated self-reporting and meticulous record-keeping within community health service centers. Concurrently, community workers, including members of neighborhood committees or health service center personnel well-versed in the local community, played an active role in the survey, assuming responsibility for the preliminary screening of potential research participants. The detailed description of this survey has been previously reported [15,19].

This study followed the tenets of the Declaration of Helsinki and received approval from the Ethics Research Committee of the Health Culture Research Center of Shaanxi (No. JKWH-2022-02). All participants were required to sign informed consent prior to participating in the study. The study was carried out anonymously to safeguard the privacy and confidentiality of the participants.

### Survey instruments

The study employed both self-developed questionnaires and standardised questionnaires to investigate the factors associated with subjective dry eye symptoms.

#### Self-developed questionnaires

The self-developed questionnaires employed in this study consisted of items that evaluated characteristics of the participants, encompassing demographic factors including age, gender, education level, smoking status, drinking alcohol, sleep time, sleep quality, having a spouse, career status, urban-rural distribution, medical insurance type, and self-rated health status (rated on a scale ranging from 1 to 100, with 1 represents “the least healthy” and 100 represents “the healthiest”). Additionally, basic family information, including family per capita monthly income and family social status (rated on a scale of 1 to 7, with 1 denoting “lowest” and 7 denoting “highest”), was collected.

#### Standardised questionnaires

##### Subjective dry eye symptoms

Participants’ subjective dry eye symptoms were measured using the Ocular Surface Disease Index-6 (OSDI-6) [20]. Each item is rated on a 5-point Likert scale, ranging from 0 (never) to 4 (constantly). The OSDI-6 yields total scores ranging from 0 to 24, with higher scores indicating greater severity of dry eye symptoms. Based on the previous study [20], dry eye symptoms are operationally defined as OSDI-6 scores of 4 or higher. The Cronbach’s α of the OSDI-6 in this study was 0.936.

##### Personality traits

Participants’ personality traits were assessed using the Big Five Inventory-10 (BFI-10) [21]. The BFI-10 encompasses five personality dimensions: extraversion, agreeableness, conscientiousness, neuroticism, and openness. Each item is rated on a 5-point Likert scale, ranging from 1 (totally disagree) to 5 (totally agree). Five reverse-scored items are rated on a scale from 1 (totally agree) to 5 (totally disagree). Higher scores reflect greater magnitudes of personality traits. Due to the limited number of items (i.e. two items per dimension) in the BFI-10, Cronbach’s α was not calculated in this study [22].

##### Health literacy

Participants’ health literacy was examined by the Health Literacy Scale-Short Form (HLS-SF) [23]. The HLS-SF is comprised of nine items. Each item is rated on a 4-point Likert scale, ranging from 0 (very difficult) to 3 (very easy). The HLS-SF yields total scores ranging from 0 to 27, with higher scores denoting greater levels of health literacy. The Cronbach’s α of the HLS-SF in this study was 0.938.

##### Depression symptoms

Participants’ depression symptoms were evaluated using the Patient Health Questionnaire-9 (PHQ-9) [24]. Each item is rated on a 4-point Likert scale, ranging from 0 (never) to 3 (nearly every day). The PHQ-9 yields total scores ranging from 0 to 27, with higher scores representing higher severity of depression symptoms. Based on previous studies [25,26], depression symptoms are operationally defined as PHQ-9 scores of 10 or higher. The Cronbach’s α of the PHQ-9 in this study was 0.921.

##### Anxiety symptoms

Participants’ anxiety symptoms were determined by the Generalized Anxiety Disorder-7 (GAD-7) [27]. Each item is rated on a 4-point Likert scale, ranging from 0 (never) to 3 (nearly every day). The GAD-7 yields total scores ranging from 0 to 21, with higher scores conveying higher severity of anxiety symptoms. Based on previous studies [28,29], anxiety symptoms are operationally defined as GAD-7 scores of 10 or higher. The Cronbach’s α of the GAD-7 in this study was 0.942.

##### Loneliness

Participants’ loneliness was measured utilising the Three-Item Loneliness Scale (T-ILS) [30]. Each item is rated on a 3-point Likert scale, ranging from 1 (never) to 3 (often). The T-ILS yields total scores ranging from 3 to 9, with higher scores reflecting higher levels of loneliness. The Cronbach’s α of the T-ILS in this study was 0.862.

##### Problematic Internet use

Participants’ problematic Internet use was assessed by the Problematic Internet Use Questionnaire Short-Form-6 (PIUQ-SF-6) [31]. Each item is rated on a 5-point Likert scale, ranging from 1 (never) to 5 (always). The PIUQ-SF-6 yields total scores ranging from 6 to 30, with higher scores denoting increased problematic use. The Cronbach’s α of the PIUQ-SF-6 in this study was 0.932.

##### Family health

Participants’ family health was appraised using the Family Health Scale-Short Form (FHS-SF) [32]. The FHS-SF is comprised of ten items. Each item is rated on a 5-point Likert scale, ranging from 1 (strongly disagree) to 5 (strongly agree). Three reverse-scored items are rated on a scale from 1 (strongly agree) to 5 (strongly disagree). The FHS-SF yields total scores ranging from 10 to 50, with higher scores indicating greater levels of family health. The Cronbach’s α of the FHS-SF in this study was 0.825.

##### Family communication

Participants’ family communication was examined using the Family Communication Scale-10 (FCS-10) [33]. Each item is rated on a 5-point Likert scale, ranging from 1 (strongly disagree) to 5 (strongly agree). The FCS-10 yields total scores ranging from 10 to 50, with higher scores representing higher levels of communication among family members. The Cronbach’s α of the FCS-10 in this study was 0.966.

Based on the socioecological model, the individual characteristics level encompassed factors including age, gender, education level, personality traits, and health literacy. At the individual behaviours level, the study considered factors including smoking status, drinking alcohol, depression symptoms, anxiety symptoms, loneliness, sleep time, sleep quality, problematic Internet use, and self-rated health status. The interpersonal networks level included having a spouse, family health, and family communication. At the community level, the study considered factors including career status, urban-rural distribution, family per capita monthly income, and family social status. The policy level included medical insurance type.

### Statistical analyses

First, the Kolmogorov-Smirnov test was utilised to assess the normality of continuous variables. A visual examination of Q-Q plots demonstrated that the distribution of continuous variables closely approximated normality. The descriptive statistics of continuous variables were summarised using the mean and standard deviation (SD), and categorical variables were depicted using numbers and percentages. Second, a random forest classification analysis was utilised to determine the importance of 26 factors derived from the socioecological model. Third, the top 50% of important factors identified from the random forest classification analysis were integrated into a univariate logistic regression model to investigate the association between the study variables and subjective dry eye symptoms. Fourth, study variables that demonstrated statistical significance at the *P* < 0.05 level in the univariate logistic model were incorporated into a multivariable logistic model for further investigation. Finally, the estimation of the network model (i.e. ocular surface disease index measure and variables statistically significant associated with subjective dry eye symptoms in the multivariable logistic regression model) was carried out using the R packages “bootnet” and “qgraph” [34].

In the network model, each variable was assigned the role of a “node”, and the interconnections among variables were considered “edges” [35]. The thickness of the edges in the network diagram corresponds to the strength of the association between nodes. Thicker edges indicate stronger associations, whereas thinner edges represent weaker associations. For the current models, the default setting for the gamma hyperparameter was set to 0.5, and the “EBICglasso” and “mgm” methods were utilised as the default approaches [36,37].

The estimation of three widely used node centrality indices was conducted using the centrality plot function provided by the “qgraph” package [36]. Node strength measures the degree of direct connectivity of a node within the network. It is determined by summing the weights of the edges that connect the specific node to other nodes in the network. Closeness measures the indirect connectivity of a node in the network. It is calculated as the reciprocal sum of the shortest path lengths between the node and all other nodes in the network. Betweenness measures the indirect connectivity of a node within the network. It is quantified by counting the number of times the node lies on the shortest path connecting two other nodes in the network. Strength centrality is given priority due to its proportionality to the degree to which a particular node uniquely accounts for the variance in its connected nodes. The centrality indices were standardised to obtain z-scores.

To assess the accuracy and stability of the observed network model, two analyses were conducted using the “bootnet” package in R [34]. The first analysis entailed estimating the stability of node centrality, specifically focusing on the strength index. This was accomplished using a case-drop bootstrap procedure with 1000 iterations. The second analysis concentrated on evaluating the confidence intervals (CIs) of the edge weights. This was carried out using a nonparametric bootstrap procedure that encompassed 1000 iterations. The stability of node strength is visually presented and evaluated using the correlation stability coefficient (CS-C), with a threshold of 0.25 or higher, and ideally exceeding 0.50 [34]. Regarding bootstrapped CIs for edge weights, larger CIs indicate lower precision in estimating edges, suggesting less reliability. Conversely, narrower CIs indicate a more reliable network with higher precision in estimating the edge weights.

In addition, differences in network properties, such as node strengths and edge weights, were assessed using bootstrapped difference tests [34]. The statistical significance of differences between two edge weights or two node centrality indices was determined using 95% CIs.

All statistical tests were two-sided, and the significance level was set at *P* < 0.05. Statistical analyses were conducted using Stata version 16.0 (StataCorp, College Station, TX, USA) and R software version 4.3.0 (R Core Team 2023, Vienna, Austria).

## RESULTS

### Descriptive statistics

This study included 21 916 participants, with 10 958 (50.00%) males and 10 958 (50.00%) females. A total of 9561 participants (43.63%) reported experiencing subjective dry eye symptoms. The mean scores for health literacy, loneliness, problematic Internet use, self-rated health status, family health, and family communication among participants were 18.55 (SD = 5.30), 4.56 (SD = 1.62), 11.72 (SD = 5.50), 73.42 (SD = 21.58), 38.65 (SD = 6.77) and 37.60 (SD = 8.17) points, respectively (**Table 1**). The mean score range of OSDI-6 items was from 0.54 to 0.83 (**Table 2**).

**Table 1:** Characteristics of participants (n = 21 916)

Variables	Value
**Individual characteristics level**	
Age in years, mean (SD)	39.43 (18.85)
Gender, n (%)	
*Male*	10 958 (50.00)
*Female*	10 958 (50.00)
Education level, n (%)	
*Primary school and below*	3412 (15.57)
*Junior high school and senior school*	8731 (39.84)
*Junior college and above*	9773 (44.59)
Personality traits (scores), mean (SD)	
*Extraversion*	6.24 (1.62)
*Agreeableness*	7.00 (1.48)
*Conscientiousness*	6.76 (1.65)
*Neuroticism*	5.73 (1.56)
*Openness*	6.46 (1.55)
Health literacy (scores), mean (SD)	18.55 (5.30)
**Individual behaviours level**	
Smoking status, n (%)	
*No*	18 658 (85.13)
*Yes*	3258 (14.87)
Drinking alcohol, n (%)	
*No*	17 362 (79.22)
*Yes*	4554 (20.78)
Depression symptoms, n (%)	
*Absence*	16 927 (77.24)
*Presence*	4989 (22.76)
Anxiety symptoms, n (%)	
*Absence*	18 808 (85.82)
*Presence*	3108 (14.18)
Loneliness (scores), mean (SD)	4.56 (1.62)
Sleep time (hours/day), n (%)	
*<5*	1228 (5.60)
*5-6*	4511 (20.58)
*6-7*	8428 (38.46)
*>7*	7749 (35.36)
Sleep quality, n (%)	
*Very bad*	612 (2.79)
*Relatively bad*	2993 (13.66)
*Relatively good*	12 143 (55.41)
*Very good*	6168 (28.14)
Problematic Internet use (scores), mean (SD)	11.72 (5.50)
Self-rated health status (scores), mean (SD)	73.42 (21.58)
**Interpersonal networks level**	
Have a spouse, n (%)	
*No*	9479 (43.25)
*Yes*	12 437 (56.75)
Family health (scores), mean (SD)	38.65 (6.77)
Family communication (scores), mean (SD)	37.60 (8.17)
**Community level**	
Career status, n (%)	
*Student*	6580 (30.02)
*Have no job*	5126 (23.39)
*Have a job*	10 210 (46.59)
Urban-rural distribution, n (%)	
*Rural*	6728 (30.70)
*Urban*	15 188 (69.30)
Family per capita monthly income (Chinese yuan), n (%)	
*≤3000*	7229 (32.99)
*3001-6000*	9026 (41.19)
*≥6001*	5661 (25.83)
Family social status (scores), mean (SD)	4.35 (1.30)
**Policy level**	
Medical insurance type, n (%)	
*Self-pay*	1669 (7.62)
*Resident basic medical insurance*	11 846 (54.05)
*Employee basic medical insurance*	3597 (16.41)
*Commercial and multiple insurances*	4804 (21.92)
Ocular surface disease index (scores), mean (SD)	4.29 (5.03)
Subjective dry eye symptoms, n (%)	
*Absence*	12 355 (56.37)
*Presence*	9561 (43.63)

SD – standard deviation

**Table 2:** Descriptive statistics of the Ocular Surface Disease Index-6 (OSDI-6) items

Item abbreviations	Item content	Item, mean (SD)	Betweenness*	Closeness*	Strength*	Predictability*
OSDI1	Eyes that are sensitive to light	0.83 (1.00)	0.221	-0.175	-1.213	0.624
OSDI2	Blurred vision	0.81 (0.97)	0.221	0.192	-0.562	0.657
OSDI3	Driving at night	0.54 (0.92)	-1.107	-0.163	-0.524	0.646
OSDI4	Watching TV (or similar tasks)	0.62 (0.94)	1.550	1.840	1.652	0.726
OSDI5	Windy conditions	0.74 (0.98)	0.221	-0.636	0.324	0.709
OSDI6	Places or areas with low humidity	0.75 (0.97)	-1.107	-1.057	0.323	0.714

OSDI – ocular surface disease index, SD – standard deviation

*The values are raw data from the network.

### Factors associated with subjective dry eye symptoms

The random forest classification analysis identified the top 13 factors associated with subjective dry eye symptoms as follows: loneliness, problematic Internet use, depression symptoms, self-rated health status, family health, anxiety symptoms, family communication, health literacy, age, sleep quality, neuroticism, sleep time, and agreeableness (**Table 3**). The results of the univariate logistic regression analysis demonstrated a significant association between the above factors and subjective dry eye symptoms (**Table 4**). The results of the multivariate logistic regression analysis indicated that higher levels of loneliness (odds ratio (OR) = 1.29; 95% CI = 1.26 to 1.32), problematic Internet use (OR = 1.08; 95% CI = 1.08 to 1.09), family communication (OR = 1.01; 95% CI = 1.00 to 1.01), the presence of depression (OR = 1.53; 95% CI = 1.39 to 1.68)) and anxiety (OR = 1.31; 95% CI = 1.17 to 1.47) symptoms and older age (OR = 1.01; 95% CI = 1.01 to 1.01) were significantly associated with the presence of subjective dry eye symptoms. Participants who reported higher levels of self-rated health status (OR = 0.99; 95% CI = 0.98 to 0.99), family health (OR = 0.97; 95% CI = 0.96 to 0.97), health literacy (OR = 0.98; 95% CI = 0.97 to 0.99) and the agreeableness personality trait (OR = 0.97; 95% CI = 0.95 to 0.99) exhibited a lower likelihood of experiencing subjective dry eye symptoms (**Table 5**).

**Table 3:** Feature importance of random forest regression

Variables	Feature importance (%)
Loneliness	26.30
Problematic Internet use	15.60
Depression symptoms	14.20
Self-rated health status	8.60
Family health	8.40
Anxiety symptoms	6.10
Family communication	5.80
Health literacy	3.30
Age	3.00
Sleep quality	2.80
Neuroticism	1.30
Sleep time	1.10
Agreeableness	0.90
Conscientiousness	0.60
Openness	0.30
Extraversion	0.30
Family social status	0.30
Have a spouse	0.20
Career status	0.20
Education level	0.20
Drinking alcohol	0.10
Gender	0.10
Medical insurance type	0.10
Family per capita monthly income	0.10
Smoking status	0
Urban-rural distribution	0

**Table 4:** Univariate analysis of associations between study variables and subjective dry eye symptoms (n = 21 916)

Variables	OR (95% CI)	*P*-value
Loneliness	1.61 (1.58-1.64)	<0.001
Problematic Internet use	1.12 (1.12-1.13)	<0.001
Depression symptoms		
*Absence*	Reference	
*Presence*	4.56 (4.25-4.88)	<0.001
Self-rated health status	0.98 (0.97-0.98)	<0.001
Family health	0.92 (0.91-0.92)	<0.001
Anxiety symptoms		
*Absence*	Reference	
*Presence*	4.96 (4.54-5.41)	<0.001
Family communication	0.95 (0.94-0.95)	<0.001
Health literacy	0.93 (0.93-0.94)	<0.001
Age	1.00 (1.00-1.01)	<0.001
Sleep quality		
*Very bad*	Reference	
*Relatively bad*	0.48 (0.39-0.59)	<0.001
*Relatively good*	0.24 (0.20-0.29)	<0.001
*Very good*	0.16 (0.13-0.19)	<0.001
Neuroticism	1.22 (1.20-1.24)	<0.001
Sleep time		
*<5*	Reference	
*5-6*	0.69 (0.61-0.79)	<0.001
*6-7*	0.42 (0.37-0.48)	<0.001
*>7*	0.36 (0.32-0.41)	<0.001
Agreeableness	0.80 (0.79-0.82)	<0.001

OR – odds ratio, CI – confidence interval

**Table 5:** Multivariate analysis of associations between study variables and subjective dry eye symptoms (n = 21 916)

Variables	OR (95% CI)	*P*-value
Loneliness	1.29 (1.26-1.32)	<0.001
Problematic Internet use	1.08 (1.08-1.09)	<0.001
Depression symptoms		
*Absence*	Reference	
*Presence*	1.53 (1.39-1.68)	<0.001
Self-rated health status	0.99 (0.98-0.99)	<0.001
Family health	0.97 (0.96-0.97)	<0.001
Anxiety symptoms		
*Absence*	Reference	
*Presence*	1.31 (1.17-1.47)	<0.001
Family communication	1.01 (1.00-1.01)	0.002
Health literacy	0.98 (0.97-0.99)	<0.001
Age	1.01 (1.01-1.01)	<0.001
*Sleep quality*		
*Very bad*	Reference	
*Relatively bad*	0.82 (0.65-1.02)	0.073
*Relatively good*	0.67 (0.54-0.83)	<0.001
*Very good*	0.53 (0.42-0.65)	<0.001
Neuroticism	1.00 (0.98-1.03)	0.700
Sleep time		
*<5*	Reference	
*5-6*	0.98 (0.84-1.14)	0.800
*6-7*	0.83 (0.72-0.95)	0.009
*>7*	0.79 (0.69-0.92)	0.002
Agreeableness	0.97 (0.95-0.99)	0.013

OR – odds ratio, CI – confidence interval

### Network structure and centrality measures analysis

**Figure 1:**
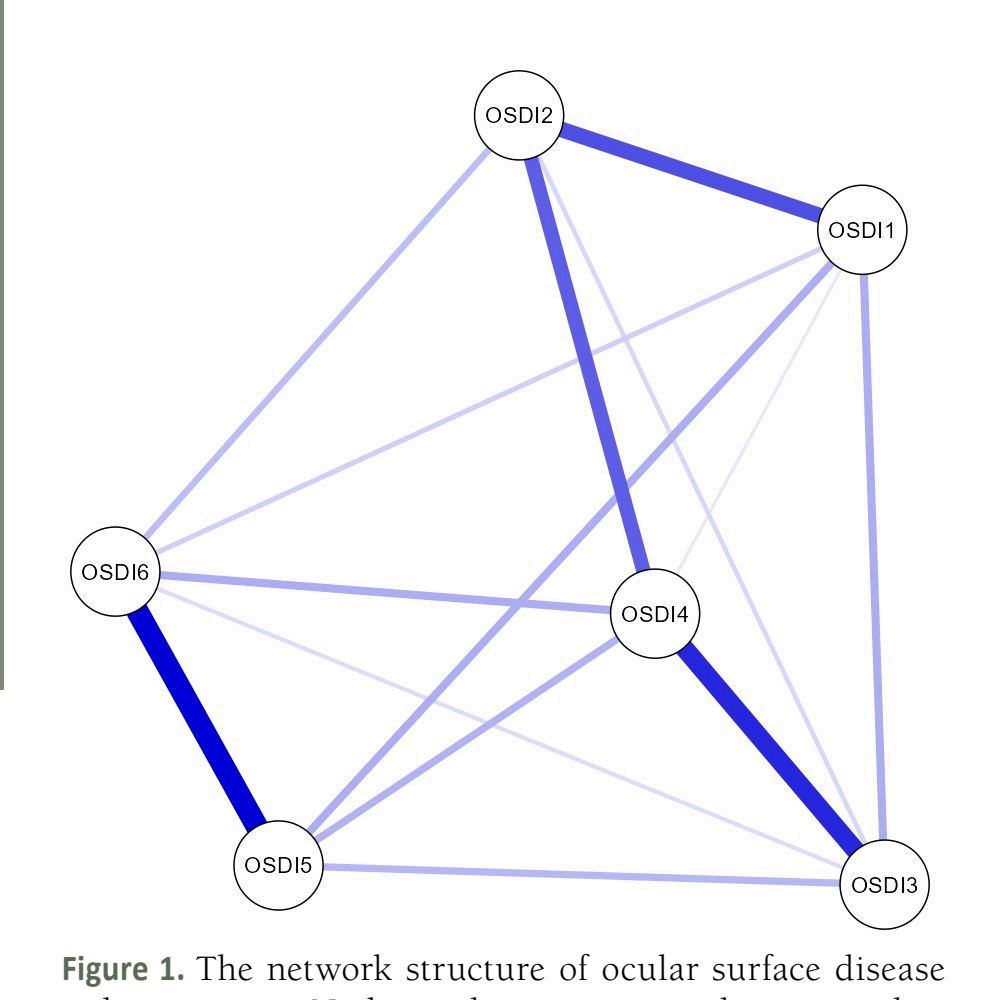
The network structure of ocular surface disease index measure. Nodes with stronger correlations tend to be closer to each other in terms of spatial proximity. The thickness of an edge serves as an indicator of the correlation strength. Positive connections are graphically depicted using blue lines. OSDI – ocular surface disease index

**Figure 2:**
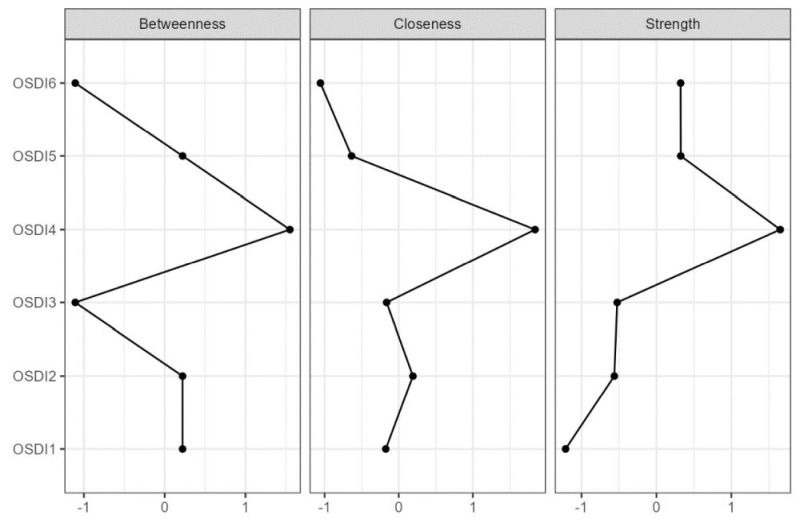
Betweenness, closeness, and strength centrality measures for the estimated network based on the data from the ocular surface disease index measure. OSDI – ocular surface disease index

The network of ocular surface disease index measures was displayed in **Figure 1**. The edge OSDI5-OSDI6 showed the strongest association. The OSDI6 item exhibited the highest node strength and had the highest predictability within the network (**Table 2**, **Figure 2**).

**Figure 3:**
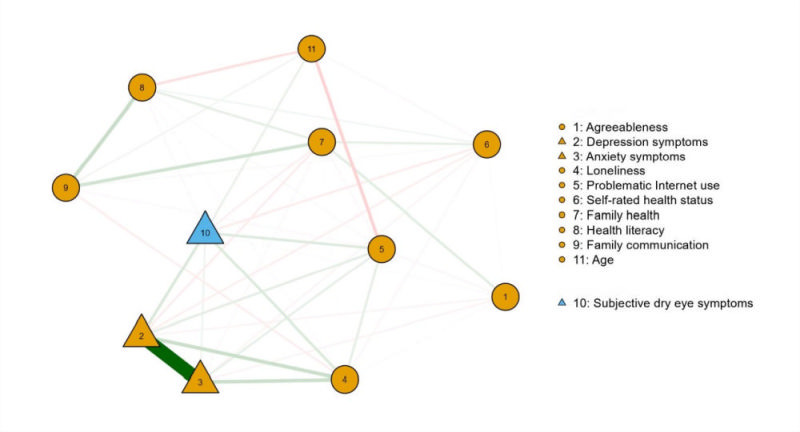
The network structure of subjective dry eye symptoms and variables significantly associated with subjective dry eye symptoms in a multivariate logistic regression analysis. Nodes with stronger correlations tend to be closer to each other in terms of spatial proximity. The thickness of an edge serves as an indicator of the correlation strength. Positive connections are graphically depicted using green lines, while negative connections are represented by red lines. Triangles symbolise categorical variables, whereas circles depict continuous variables.

The network of variables (i.e. subjective dry eye symptoms, loneliness, problematic Internet use, family communication, depression symptoms, anxiety symptoms, age, self-rated health status, family health, health literacy, and the agreeableness personality trait) was shown in **Figure 3**. Depression symptoms showed the highest node strength in the network (Table S1 and Figure S1 in the **Online Supplementary Document**).

### Network accuracy, stability, edge weight and strength centrality differences

The case-dropping bootstrap procedure (n = 1000) demonstrated that the CS-Cs for node strength were 0.75, indicating that 75% of the samples could be dropped while maintaining stability (Figure S2-3 in the **Online Supplementary Document**). The nonparametric bootstrap procedure (n = 1000) revealed statistically significant differences in the majority of comparisons involving edge weights and node strength (Figures S4-7 in the **Online Supplementary Document**). The trustworthiness of the edges was suggested by the narrow bootstrapped 95% CIs shown in Figure S8-9 in the **Online Supplementary Document**.

## DISCUSSION

To the best of our knowledge, this was the first investigation examining subjective dry eye symptoms and the associated factors among the general population in China during the COVID-19 pandemic, conducted through a nationwide survey. The study found that item 4 from the OSDI-6 questionnaire had the highest node strength in the network and identified several factors that were associated with subjective dry eye symptoms based on the socioecological model.

Our study findings indicated that the item “have problems with eyes limited in performing watching TV (or similar tasks)” from the OSDI-6 questionnaire exhibited the highest node strength within the network among the general population during the COVID-19 pandemic. The observed prominence of this item can be interpreted within the context of the COVID-19 pandemic. During the pandemic era, various public health measures, such as lockdowns and social distancing, were implemented to mitigate the spread of the virus [38]. Consequently, individuals spent more time at home and relied heavily on screens for work, education, and entertainment [39]. The increased screen time likely contributed to visual discomfort and eye-related problems [40], leading to higher endorsement of the mentioned item within the OSDI-6 questionnaire. Additionally, prolonged screen exposure during the pandemic might have resulted in visual fatigue. The human visual system is not adapted to long hours of screen usage, as it involves continuous focusing and refocusing, increased exposure to blue light, and reduced blinking frequency [41]. These factors can cause symptoms like eye strain, dryness, irritation, and blurred vision [42]. Thus, individuals experiencing such issues were more likely to endorse the item in question, reflecting the impact of increased screen time on their visual well-being. The identification of this item as having the highest node strength underscores the specific challenges faced by individuals during the COVID-19 pandemic. These findings highlight the importance of addressing visual protection during times of prolonged screen usage, such as the COVID-19 pandemic, and emphasise the need for measures to mitigate the potential negative effects on individuals’ eye health.

This study identified factors associated with subjective dry eye symptoms employing the socioecological model, offering a basis for the development of tailored promotion strategies. Specifically, at the individual characteristics level, age, agreeableness personality trait and health literacy showed associations with subjective dry eye symptoms. Additionally, at the individual behaviour level, depression symptoms, anxiety symptoms, loneliness, problematic Internet use, and self-rated health status were found to be associated with subjective dry eye symptoms. Moreover, at the interpersonal network level, family health and family communication were also identified as factors associated with subjective dry eye symptoms.

Our study revealed that participants who reported higher levels of loneliness, as well as those with the presence of depression and anxiety symptoms, were more likely to experience subjective dry eye symptoms. Several biological plausibility mechanisms could elucidate these underlying associations. First, loneliness is a complex psychosocial construct, characterised by feelings of social isolation and a lack of meaningful social connections [43]. The potential association between loneliness and subjective dry eye symptoms may be mediated by the stress response. Chronic loneliness has been associated with dysregulated stress responses, including heightened activation of the hypothalamic-pituitary-adrenal axis and increased release of stress hormones such as cortisol [44]. This heightened stress response may have a direct impact on the ocular surface, leading to changes in tear film composition and stability [45]. Additionally, lonely individuals may engage in maladaptive health behaviours, such as poor diet and lack of physical activity, which can indirectly influence subjective dry eye symptoms through systemic inflammation and oxidative stress [43,46]. Second, depression and anxiety are known to be associated with alterations in the autonomic nervous system, including increased sympathetic activity and decreased parasympathetic tone [47]. These autonomic changes can impact tear production and ocular surface health [48]. Moreover, the systemic inflammation and immune dysregulation often observed in individuals with depression and anxiety could indirectly affect the ocular surface, contributing to subjective dry eye symptoms [49]. An in-depth examination of these mechanisms, possibly through the assessment of specific inflammatory markers and autonomic function, would strengthen the rationale behind the observed associations. These findings underscore the importance of a holistic approach to ocular health. Ophthalmic care providers should consider assessing the psychological well-being of patients presenting with dry eye symptoms. By addressing not only the ocular manifestations but also the underlying psychosocial determinants, clinicians can develop more tailored and effective treatment strategies [50]. Furthermore, this approach may promote better patient outcomes and satisfaction with their eye care. From a clinical perspective, incorporating psychological support and interventions into eye care practice could be a promising avenue to explore. Moreover, interdisciplinary collaboration between ophthalmologists, psychologists, and other health care professionals can facilitate a comprehensive approach to ocular health, ensuring that patients receive well-rounded care that addresses both the physical and emotional aspects of their condition [51]. On a broader scale, these findings have implications for public health initiatives. Dry eye symptoms are prevalent conditions that can significantly affect individuals’ quality of life, particularly as the global population continues to age [1]. Recognising the role of psychosocial factors in dry eye symptoms highlights the need for public health campaigns and policies that promote social connectedness, mental health, and overall well-being. Initiatives aimed at reducing loneliness and addressing mental health issues could indirectly contribute to the prevention and management of dry eye symptoms at the population level.

The results of our study confirmed positive associations between subjective dry eye symptoms and both problematic Internet use and family communication, corroborating existing studies [8,52]. The COVID-19 pandemic has necessitated a significant shift in daily activities, with increased reliance on digital platforms for work, education, and social interactions [39]. This shift has led to a rise in problematic Internet use, encompassing behaviours such as excessive social media consumption, problematic online gaming, or compulsive Internet browsing [53]. Prolonged screen time often leads to decreased blink rate and incomplete blinking, which, in turn, reduces the even distribution of tears over the ocular surface [54]. This disruption of the tear film can result in increased tear evaporation and an unstable tear film, contributing to ocular discomfort [55]. Furthermore, exposure to the blue light emitted by digital screens can disrupt circadian rhythms and affect the synthesis of melatonin, a hormone with known anti-inflammatory and antioxidant properties [56]. This disturbance in circadian rhythms and melatonin production may disrupt ocular homeostasis, exacerbating dry eye symptoms. Simultaneously, the COVID-19 pandemic has compelled individuals to spend more time together as families due to stay-at-home orders and limited social interactions [57]. While increased family communication can be beneficial for emotional well-being and support, it can also give rise to conflicts and misunderstandings [58]. The prolonged proximity and heightened emotional states may lead to disagreements, tensions, and misunderstandings within family dynamics [59]. These interpersonal challenges, coupled with the overall stress brought about by the pandemic, can contribute to the development or exacerbation of subjective dry eye symptoms [60]. Elevated stress levels can stimulate the sympathetic nervous system, leading to decreased tear production and increased tear evaporation [44]. Chronic stress can also promote the release of pro-inflammatory cytokines, which may exacerbate ocular surface inflammation and contribute to dry eye symptoms [45]. These findings underscore the novel strategies that ophthalmologists may employ in the diagnosis and treatment of dry eye symptoms within clinical practice. Specifically, a proactive inquiry into patients’ internet usage habits and family communication dynamics is recommended to identify potential factors associated with dry eye symptoms. Such personalised assessments can contribute to a more comprehensive deconstruction of the etiology and progression mechanisms of the condition, ultimately facilitating the provision of more precise therapeutic recommendations [61]. These recommendations encompass advising on the limitation of excessive digital screen exposure and providing strategies for enhancing family communication, offering a comprehensive approach aimed at improving the efficacy of dry eye syndrome treatment. In the field of ocular care, these findings reveal potential risks to ocular health associated with internet usage. Consequently, ocular care professionals and stakeholders in the industry should actively formulate forward-looking measures to mitigate potential harms arising from prolonged digital screen exposure. These measures may encompass widespread public education emphasising the importance of moderate digital screen usage and regular ocular rest. Additionally, the exploration of technology interfaces and products designed to be more eye-friendly represents a noteworthy avenue for development. From a public health perspective, these findings suggest the need for comprehensive measures to manage problematic internet use while promoting family communication, with the aim of reducing the incidence of dry eye symptoms. This could involve the implementation of extensive health education campaigns, advocating for healthy digital media consumption habits, and encouraging positive family communication practices [62]. These efforts can help mitigate potential health risks associated with excessive internet use and social isolation. Furthermore, with the advancement of digitalisation and the use of intelligent terminals, people’s screen time is gradually extended, which is not only limited to China, but also global [5]. Public health policies and advocacy organisations should consider incorporating these discoveries into their frameworks for health promotion and policy development, thereby enhancing public awareness and facilitating the implementation of appropriate interventions.

Our study findings indicated that participants with higher levels of health literacy, better family health, and superior self-rated health status are less likely to experience subjective dry eye symptoms. Health literacy plays a pivotal role in equipping individuals with the knowledge and skills necessary to comprehend ocular health information, make informed decisions, and adopt preventive measures [63]. During the pandemic, when individuals are exposed to prolonged screen time and potential eye strain, higher health literacy may empower individuals to engage in protective behaviours, such as implementing proper visual ergonomics, practicing regular eye exercises, and adhering to recommended screen breaks [64]. Consequently, individuals with enhanced health literacy are better equipped to manage and prevent subjective dry eye symptoms. Moreover, family health acts as a supportive framework within which individuals can cultivate healthy habits and behaviours [65]. Families that prioritise health and well-being create an environment conducive to the adoption of protective measures against subjective dry eye symptoms. Through encouraging behaviours like balanced screen usage, promoting eye hygiene practices, and fostering a positive atmosphere that addresses stress management, families contribute to the overall ocular health of their members [66]. Furthermore, individuals with superior self-rated health status demonstrate a subjective perception of their well-being, encompassing physical and mental aspects [67]. This positive self-assessment may reflect a proactive approach to health management, including ocular health. Individuals of this nature are likely to engage in behaviours that protect their overall health, such as maintaining a healthy lifestyle, practicing self-care and seeking appropriate eye care when needed. Consequently, their reduced likelihood of experiencing subjective dry eye symptoms can be attributed to their proactive and holistic approach to health. Given the COVID-19 pandemic’s impact on increased screen time and the associated risks of subjective dry eye symptoms [68], our study highlights the importance of health literacy, family health, and self-rated health status as protective factors. Healthcare professionals should emphasise the significance of health literacy, provide educational resources to enhance ocular health awareness and promote healthy family dynamics. By empowering individuals with knowledge, fostering supportive family environments, and encouraging a proactive approach to health, health care professionals can contribute to the prevention and management of subjective dry eye symptoms during these unprecedented times.

Our study findings suggested a positive association between older age and the presence of subjective dry eye symptoms. While it is recognised that ocular discomfort and eye strain can affect individuals of all age groups, the impact of aging on ocular health is particularly noteworthy, as it is characterised by a multitude of structural and functional alterations within the ocular system [69]. Specifically, the aging process is marked by changes such as reduced tear production, alterations in tear film stability, and decreased corneal sensitivity, all of which contribute to the development of dry eye symptoms, including ocular discomfort, redness, and visual disturbances [70,71]. These age-related changes create a vulnerable ocular environment, rendering older individuals more prone to the adverse effects of external factors, including the extended screen time necessitated by the COVID-19 pandemic [6]. As older individuals often experience age-related ocular changes, the combined impact of these alterations and heightened screen exposure during the pandemic can further exacerbate dry eye symptoms in this population. Moreover, older adults may have a higher prevalence of comorbidities, including conditions such as diabetes, hypertension, and autoimmune disorders, which are known to be associated with an increased risk of dry eye syndrome [9]. These underlying health conditions, prevalent among older individuals, can contribute to the development and severity of subjective dry eye symptoms. In addition to these factors, the physiological changes within the ocular system that accompany aging are worth considering. The decline in tear film quality and stability, attributed to the reduction in the production of the aqueous layer, decreased functionality of meibomian glands, and alterations in tear film composition, results in an unstable tear film that inadequately protects the ocular surface [72]. This instability, in turn, increases the susceptibility to desiccation and irritation, fundamental components of dry eye symptoms. Furthermore, aging often leads to a systemic decline in cellular and tissue functions, which can have a profound impact on the ocular environment. For example, corneal epithelial cells may exhibit reduced regenerative capacity and increased susceptibility to environmental stressors, rendering the corneal surface more susceptible to damage [73]. In addition, age-related changes in the autonomic nervous system, particularly the parasympathetic innervation of the lacrimal glands, can result in decreased tear production and an impaired ability to respond to environmental stimuli that trigger reflex tearing [74]. Consequently, older individuals may have a diminished capacity to alleviate dry eye symptoms under challenging conditions. From a clinical standpoint, these findings emphasise the need for health care providers to be vigilant in recognising and managing dry eye symptoms in older patients. Dry eye symptoms, if left untreated, can result in discomfort and a decreased quality of life [3]. Therefore, ophthalmologists and primary care physicians must be well-versed in the multifaceted nature of the dry eye, particularly in older individuals who may be at a higher risk due to age-related ocular changes and comorbidities. Regular screening and early intervention should be integral components of clinical practice to mitigate the progression and severity of subjective dry eye symptoms in this demographic [75]. Moreover, eye care specialists should tailor their treatment approaches to account for the unique challenges faced by older patients. This might involve the use of advanced diagnostic tools to assess tear film quality, personalised treatment plans that address age-related changes in the ocular environment, and education on the management of dry eye symptoms [76]. The findings also underscore the importance of interdisciplinary collaboration, as the influence of systemic comorbidities on dry eye highlights the need for coordination between ophthalmologists and specialists in fields such as endocrinology, cardiology, and immunology. Furthermore, the insights provided by this research have far-reaching implications for public health initiatives. With the global increase in life expectancy, the prevalence of age-related ocular conditions, including dry eye, is likely to rise [2]. Public health campaigns should focus on raising awareness of the ocular health challenges faced by older individuals and promoting preventive measures. These initiatives might include advocating for regular eye examinations in the elderly population, educating the public about the importance of maintaining ocular health through lifestyle modifications, and addressing the digital eye strain exacerbated by the COVID-19 pandemic [75]. As populations age across the world, health care systems and policies must adapt to accommodate the specific needs of older individuals, including their ocular health. By understanding the interplay of age-related changes and environmental factors, countries can implement proactive strategies to alleviate the burden of dry eye symptoms in their aging populations.

In addition, our study found that participants exhibiting agreeableness personality traits demonstrate a decreased likelihood of experiencing subjective dry eye symptoms. One conceivable avenue through which agreeableness could influence dry eye symptoms is the modulation of stress response. Agreeable individuals, characterised by their propensity to be cooperative, empathetic, and harmonious in social situations, may exhibit more adaptive coping strategies and a greater ability to manage stressors effectively [77]. Chronic stress is recognised as a risk factor for the development and exacerbation of dry eye symptoms, as it can lead to hormonal imbalances and systemic inflammation, both of which can affect the ocular surface [46]. The reduced stress levels and enhanced coping mechanisms in individuals with high agreeableness traits may confer a protective effect against the development of dry eye symptoms [45]. Moreover, agreeable individuals tend to prioritise the well-being of others and exhibit a greater tendency toward engaging in health-promoting behaviours [78]. This propensity may extend to their ocular health practices, leading to greater adherence to recommended ocular hygiene routines, such as regular eye hygiene maintenance, adequate blink frequency, and the maintenance of a balanced ocular environment. As a result, their proactive engagement in these practices may contribute to the prevention or mitigation of dry eye symptoms. Additionally, agreeableness has been associated with individual differences in neurophysiological processes, including altered stress response systems, enhanced emotion regulation abilities, and increased activity in brain regions involved in social cognition [79]. These neurobiological factors could influence ocular health by improving the regulation of tear film dynamics, ocular surface homeostasis, and the perception of ocular discomfort [80]. Such neurobiological mechanisms may underlie the decreased vulnerability to the development of dry eye symptoms in individuals with high agreeableness traits. Furthermore, agreeable individuals, owing to their harmonious social interactions, tend to maintain more extensive and robust social networks, fostering social support and interpersonal relationships [81]. This psychosocial aspect of agreeableness can influence ocular health through mechanisms such as stress reduction, as social support is known to mitigate the negative impact of stress [82]. Additionally, social connectedness may positively affect lifestyle factors such as regular physical activity, which can have a beneficial influence on tear film stability and ocular surface health [83]. This finding emphasises the significance of incorporating personality traits into ocular health research, as it sheds light on the association between agreeableness and subjective dry eye symptoms. From a clinical perspective, recognising the influence of personality traits on ocular health allows health care providers to adopt a more holistic approach to patient care. Assessing an individual’s personality traits, including agreeableness, can provide valuable insights into their susceptibility to dry eye symptoms and their likelihood of adhering to recommended ocular hygiene practices. Tailoring treatment and management strategies to align with an individual’s personality profile can improve patient outcomes and overall satisfaction. This personalised approach to care is especially relevant in an era of patient-centered health care and precision medicine [61]. Furthermore, eye care specialists can consider integrating personality assessments into patients’ diagnostic protocols, complementing traditional clinical evaluations. By doing so, individuals can gain a deeper understanding of the psychosocial and behavioural factors that may contribute to dry eye symptoms. Such a holistic approach to patient care is likely to enhance the patient-doctor relationship and improve overall treatment outcomes, ultimately contributing to the well-being of patients [84]. Additionally, the implications of these findings extend to public health initiatives. Recognising the influence of personality traits, such as agreeableness, on ocular health underscores the need for public health campaigns and educational programmes that raise awareness about the psychosomatic dimensions of ocular health. These initiatives can advocate for regular eye examinations that consider personality profiles, educate the public about the importance of personalised ocular health practices, and promote the importance of a well-balanced and supportive social environment for ocular health [85]. On a broader scale, understanding the impact of personality traits on ocular health has global relevance. As populations age worldwide, the prevalence of ocular conditions, including dry eye, is likely to increase [1]. Recognising the interplay of personality traits and ocular health and addressing it comprehensively can serve as a model for global health initiatives that promote patient-centered care and well-being. The findings highlight the importance of considering individual differences in ocular health practices, emphasising the significance of a personalised and holistic approach to ocular health.

Despite this study being grounded in a socioecological model and encompassing several variables, factors such as individual characteristics level including ocular surface characteristics and systemic diseases, individual behaviours level including the use of electronic devices, and community level including humidity and air quality were not comprehensively integrated into this study. Prior study has indicated that individuals lacking access to electricity tend to exhibit a reduced prevalence of dry eye symptoms [86]. This phenomenon has been attributed, in part, to the limited use of electronic devices, such as smartphones and computers [42]. Electronic devices, with their bright screens and prolonged usage, have been identified as potential contributors to dry eye symptoms due to factors such as increased screen time, reduced blink rate, and decreased tear film stability [40]. Consequently, the absence of electricity indirectly limits the exposure to these devices, offering a biologically plausible explanation for the observed protective effect on dry eye symptoms. In addition, previous research has examined various variables that could be biologically plausible in their association with subjective dry eye symptoms [9,87,88]. These variables encompass environmental conditions, such as humidity and air quality, ocular surface characteristics, and systemic diseases. For instance, low humidity and poor air quality have been linked to increased ocular discomfort [87]. Factors related to the ocular surface, including tear film stability, blink rate, and meibomian gland function, have been investigated as contributors to dry eye symptoms [88]. Additionally, systemic conditions like diabetes and autoimmune disorders may manifest as dry eye symptoms [9], highlighting the importance of comprehensively understanding the interplay of these variables in the etiology of subjective dry eye symptoms.

The limitations of this study deserve mention. First, this study used OSDI-6 to evaluate subjective dry eye symptoms, rather than relying on objective clinical appraisal or diagnostic criteria. Future research should contemplate the utilisation of objective clinical evaluations or diagnostic criteria. Second, our study was unable to establish causal associations between variables due to the cross-sectional design. Future research should prioritise longitudinal studies that can investigate dynamic networks and identify the variables that have effects on subjective dry eye symptoms. Third, considering the elevated awareness and concerns surrounding health in the context of the COVID-19 pandemic, there is a potential for an overestimation of responses to inquiries regarding health-related aspects, including health literacy and family health. Fourth, limited by the data elements collected, this study did not investigate the participants’ history of ocular diseases or whether they had received relevant ophthalmic treatments. Finally, in this study, factors potentially associated with subjective dry eye symptoms, such as the use of electronic devices, biological factors like humidity and air quality, ocular surface characteristics, and systemic diseases, were not included. Future prospective studies should incorporate comprehensive assessments of these variables to elucidate the intricate interactions that contribute to the development of dry eye symptoms, thus providing a more comprehensive understanding of the multifaceted etiology of this condition.

## CONCLUSIONS

This study identified associations between subjective dry eye symptoms and factors within the framework of the multilevel socioecological model. Additionally, it revealed that the item “watching TV (or similar tasks)” exhibited the highest node strength within the network during the COVID-19 pandemic. These results provide valuable insights into the role of prolonged visual activities in the development and exacerbation of subjective dry eye symptoms. Additionally, this study contributes to the existing knowledge base by shedding light on the complex associations between subjective dry eye symptoms and some factors such as loneliness and problematic Internet use, guiding future interventions and initiatives in addressing these factors on the occurrence of dry eye symptoms.

## Additional material


Online Supplementary Document
