# Supplementary material for: Subjective dry eye symptoms and associated factors among the national general population in China during the COVID-19 pandemic: A network analysis
Source: J Glob Health. 2023 Dec 1;13:06052. doi: 10.7189/jogh.13.06052 (PMC10691301; doi:10.7189/jogh.13.06052)
Supplement: Online Supplementary Document [file jogh-13-06052-s001.pdf]

Table S1. Centrality measures for subjective dry eye symptoms and variables significantly associated with subjective dry eye symptoms in a multivariate logistic regression analysis.

Variables	Network		
	Betweenness	Closeness	Strength
Agreeableness	-1.094	-2.311	−1. 113
Depression symptoms	1.312	1.207	2.084
Anxiety symptoms	-0.793	0.771	1.705
Loneliness	-0.492	0.471	0.046
Problematic Internet use	-0.191	0.127	-0.120
Self-rated health status	-1.094	-1.039	-0.753
Family health	1.914	0.724	-0.178
Health literacy	0.109	-0.504	-0.474
Family communication	1.012	0.234	-0.437
Subjective dry eye symptoms	-0.492	0.610	-0.072
Age	-0.191	-0.290	-0.689

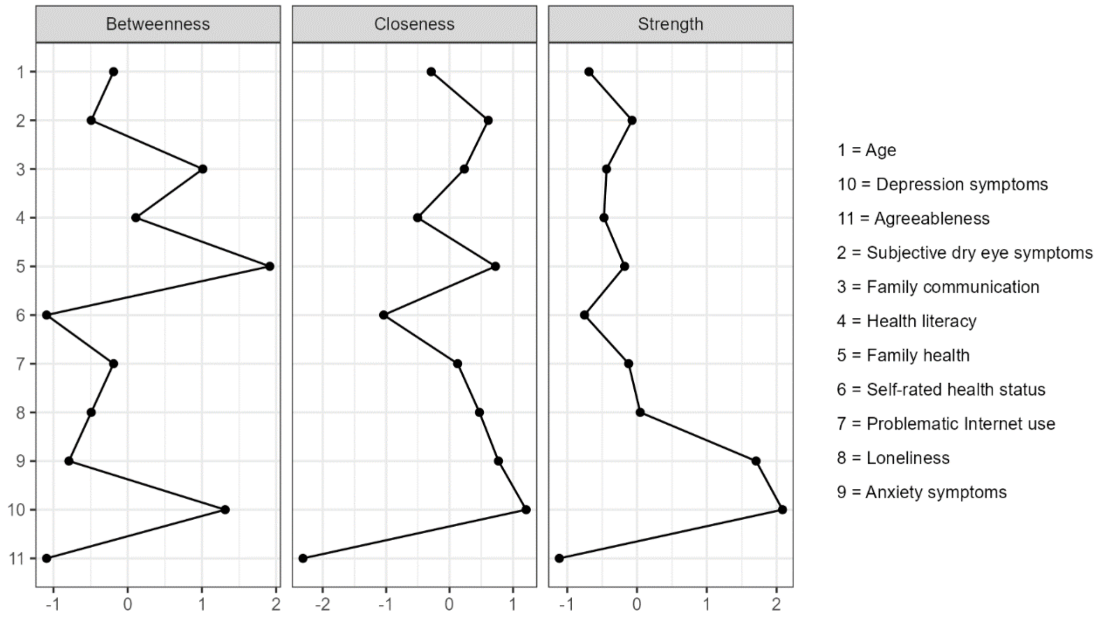

Figure S1. Betweenness, closeness, and strength centrality measures for the estimated network of subjective dry eye symptoms and variables significantly associated with subjective dry eye symptoms in a multivariate logistic regression analysis.

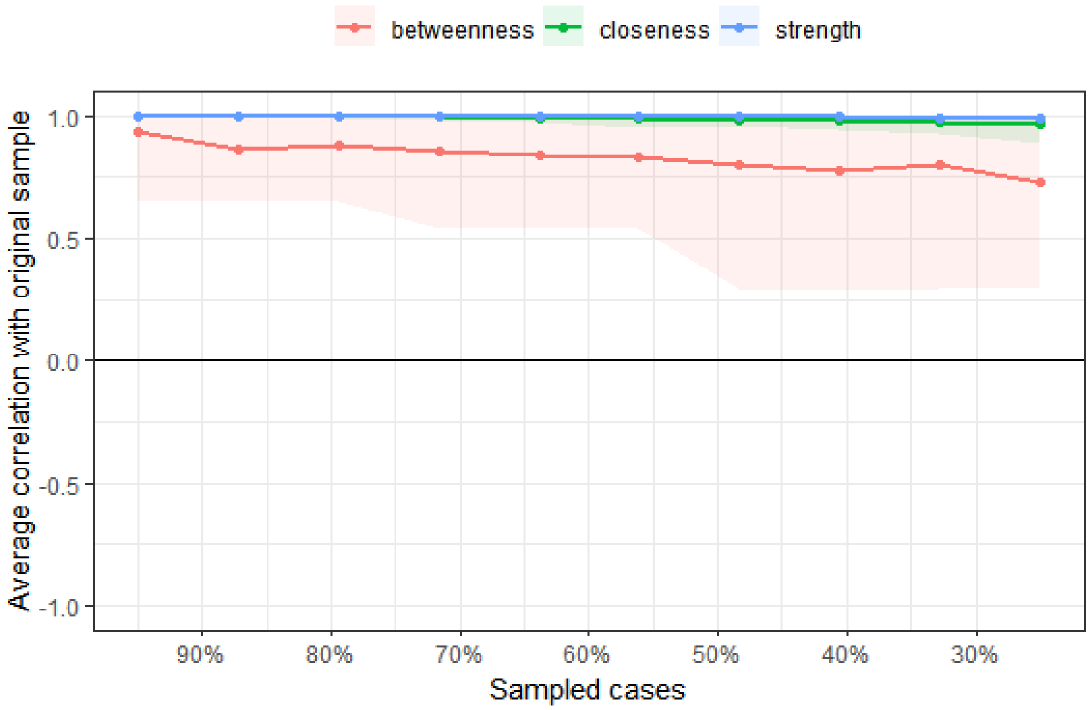

Figure S2. Stability of the centrality indices for the estimated network of ocular surface disease index measure. The x-axis illustrated the percentage of cases from the original sample at each stage. The y-axis illustrated the average correlations between centrality indices extracted from the original network and those recalculated for networks from the increasing percentage of cases that have been excluded. Each line revealed the correlations among betweenness, closeness, and strength.

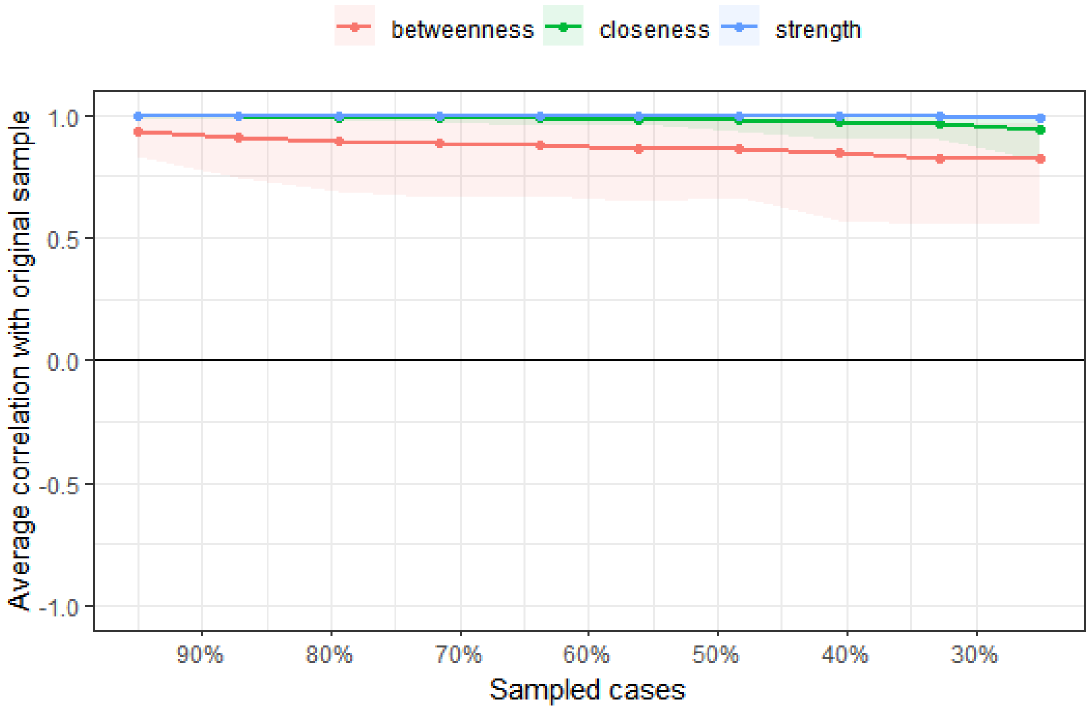

Figure S3. Stability of the centrality indices for the estimated network of subjective dry eye symptoms and variables significantly associated with subjective dry eye symptoms in a multivariate logistic regression analysis. The x-axis illustrated the percentage of cases from the original sample at each stage. The y-axis illustrated the average correlations between centrality indices extracted from the original network and those recalculated for networks from the increasing percentage of cases that have been excluded. Each line revealed the correlations among betweenness, closeness, and strength.

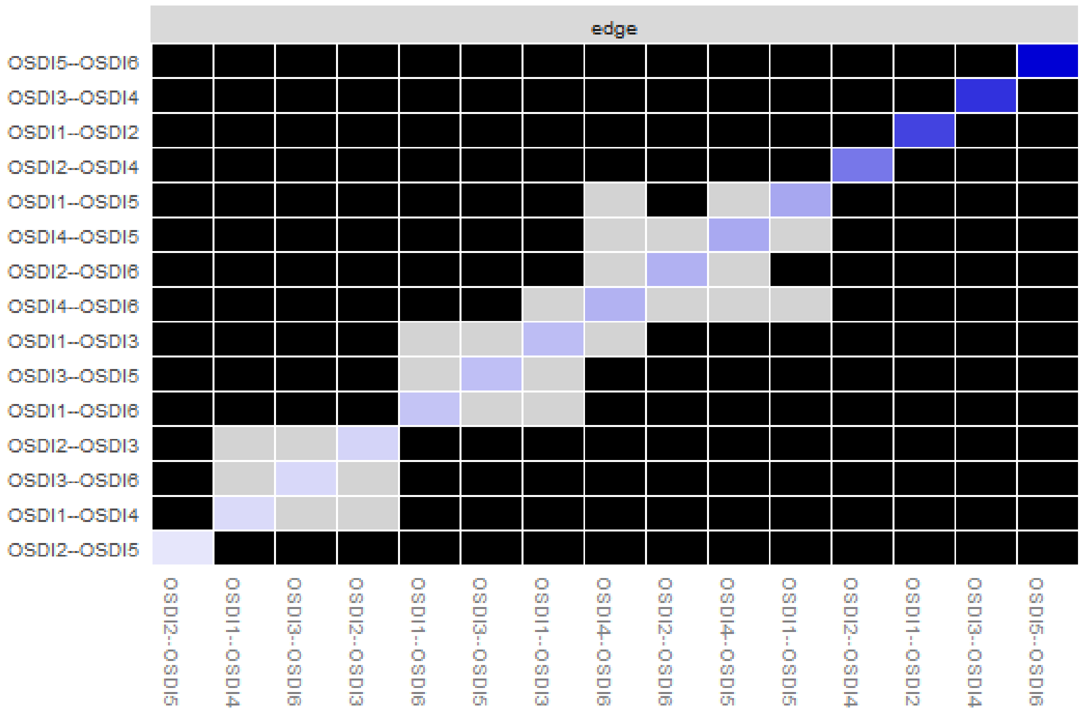

Figure S4. Statistical difference tests for edge weight. The results are based on the network structure of the ocular surface disease index measure. The figure presents the results of bootstrapped difference tests (α=0.05) conducted on edge weights. The color of the boxes signifies whether the edge weights exhibit significant differences (black) or lack significant differences (gray) from each other. The diagonal line indicates the strength of the edge weights, transitioning from red (indicating negative associations) to white (representing weaker edges), and ultimately blue (representing stronger edge weights).

Abbreviation: OSDI, ocular surface disease index.

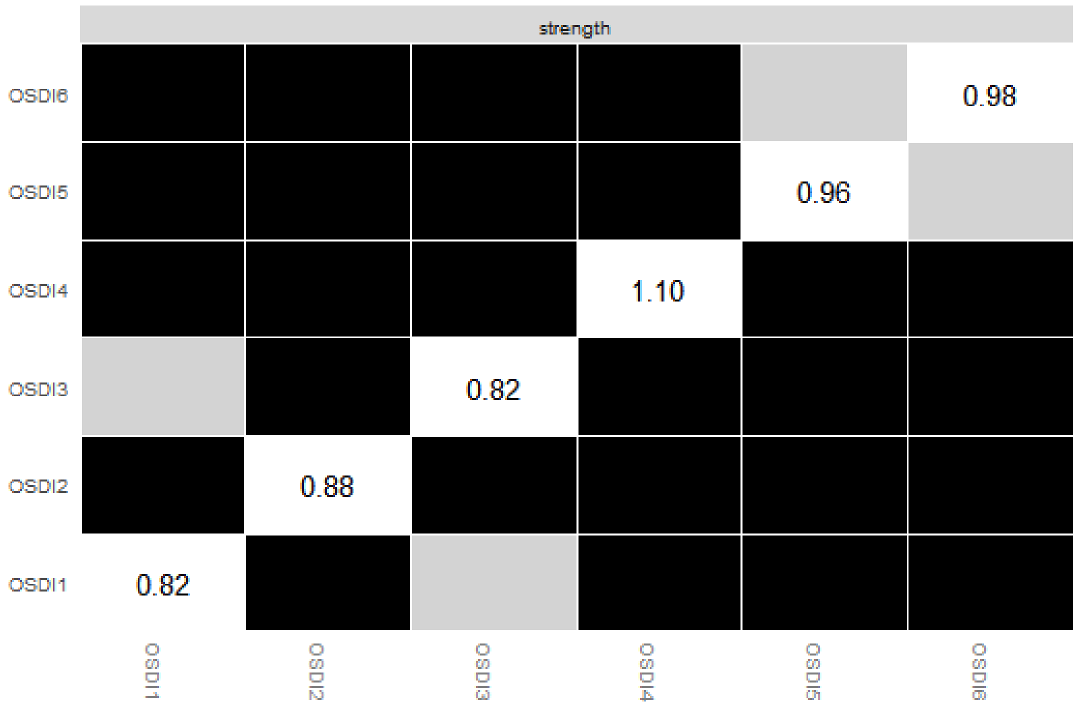

Figure S5. Statistical difference test for node strength. The results are based on the network structure of the ocular surface disease index measure. The figure presents the stability difference tests (α=0.05) conducted for "node strength". The color of the boxes represents the presence of significant differences between symptoms: gray boxes indicate no significant differences, while black boxes indicate significant differences. The value of node strength for a specific node is indicated by the number within the white boxes, corresponding to the positions along the diagonal line.

Abbreviation: OSDI, ocular surface disease index.

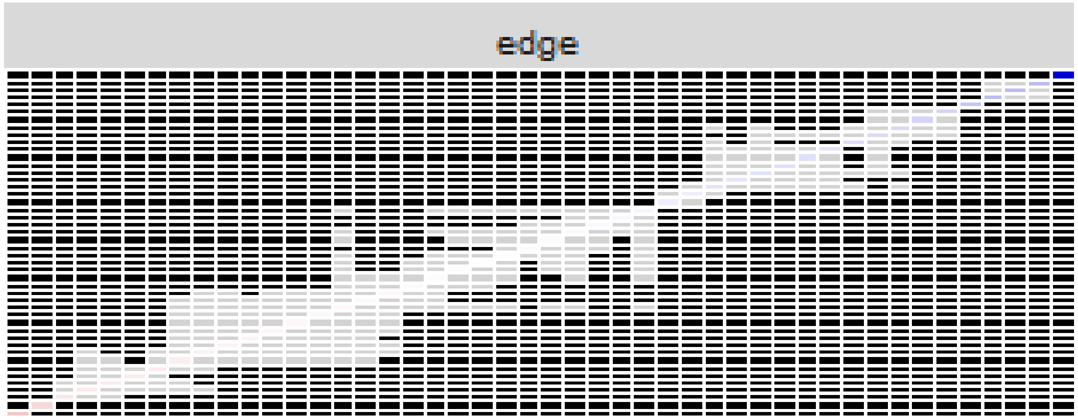

Figure S6. Statistical difference tests for edge weight. The results are based on the

network structure of subjective dry eye symptoms and variables significantly associated with subjective dry eye symptoms in a multivariate logistic regression analysis. The figure presents the results of bootstrapped difference tests (α=0.05) conducted on edge weights. The color of the boxes signifies whether the edge weights exhibit significant differences (black) or lack significant differences (gray) from each other. The diagonal line indicates the strength of the edge weights, transitioning from red (indicating negative associations) to white (representing weaker edges), and ultimately blue (representing stronger edge weights). To ensure a clear and uncluttered visualization, the x-axis and y-axis labels have been omitted.

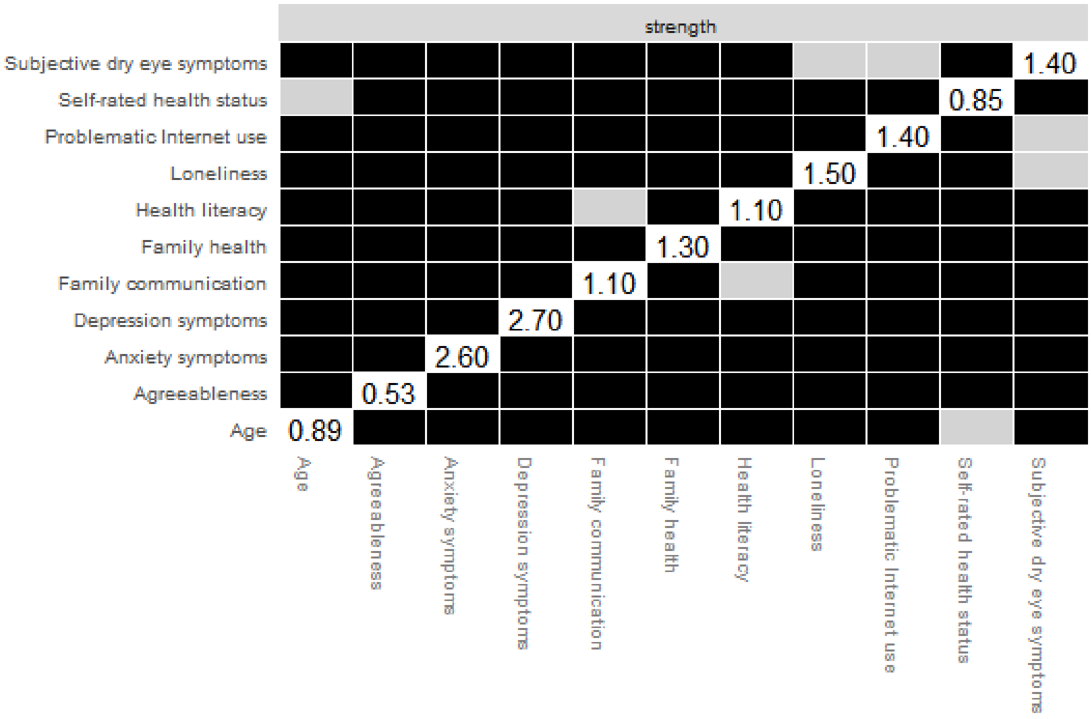

Figure S7. Statistical difference test for node strength. The results are based on the network structure of subjective dry eye symptoms and variables significantly associated with subjective dry eye symptoms in a multivariate logistic regression analysis. The figure presents the stability difference tests (α=0.05) conducted for "node strength". The color of the boxes represents the presence of significant differences between symptoms: gray boxes indicate no significant differences, while black boxes indicate significant differences. The value of node strength for a specific node is indicated by the number within the white boxes, corresponding to the positions along the diagonal

line.

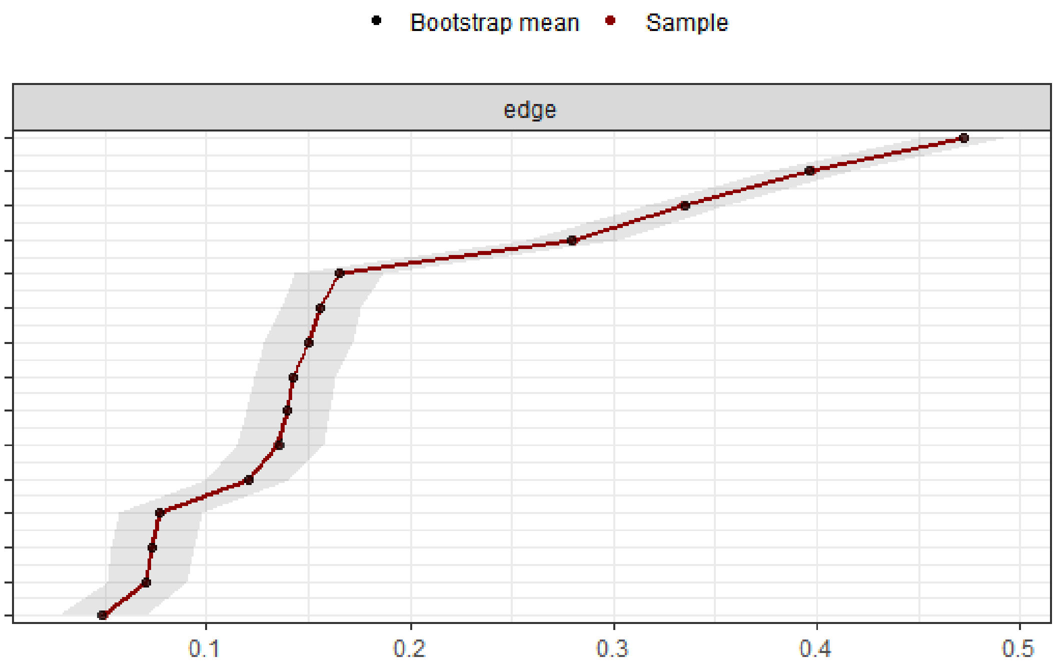

Figure S8. Ninety-five percent confidence intervals around the edge weights of the ocular surface disease index measure. The sample values are represented by the red line, while the gray area depicts the bootstrapped confidence intervals. Each horizontal line corresponds to an edge within the network, arranged in descending order based on the edge weight, ranging from the highest to the lowest. In situations where ties arise (such as when multiple edge weights are precisely estimated as 0), the edges are arranged based on the mean value derived from the bootstrap samples. To ensure a clear and uncluttered visualization, the x-axis and y-axis labels have been omitted.

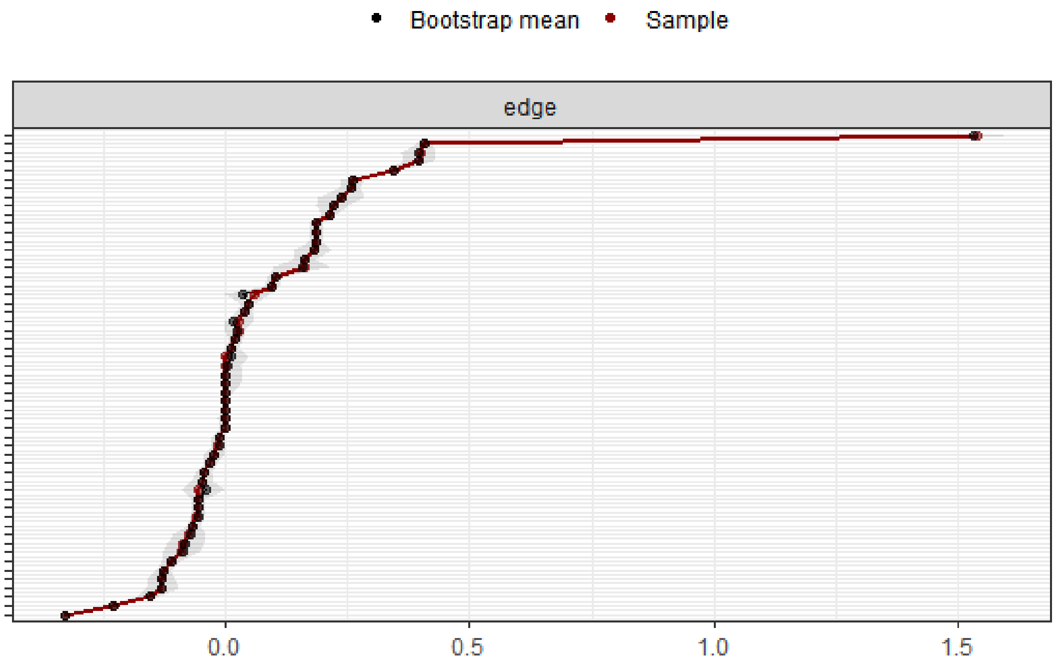

Figure S9. Ninety-five percent confidence intervals around the edge weights of subjective dry eye symptoms and variables significantly associated with subjective dry eye symptoms in a multivariate logistic regression analysis. The sample values are represented by the red line, while the gray area depicts the bootstrapped confidence intervals. Each horizontal line corresponds to an edge within the network, arranged in descending order based on the edge weight, ranging from the highest to the lowest. In situations where ties arise (such as when multiple edge weights are precisely estimated as 0), the edges are arranged based on the mean value derived from the bootstrap samples. To ensure a clear and uncluttered visualization, the x-axis and y-axis labels have been omitted.
